# The influence of a biopsychosocial-based treatment approach to primary overt hypothyroidism: a protocol for a pilot study

**DOI:** 10.1186/1745-6215-11-106

**Published:** 2010-11-15

**Authors:** Benjamin T Brown, Rod Bonello, Henry Pollard, Petra Graham

**Affiliations:** 1 Department of Chiropractic, Macquarie University, NSW, 2109 Australia; 2 Department of Statistics, Macquarie University, NSW, 2109 Australia

## Abstract

**Background:**

Hypothyroidism is a prevalent endocrine condition. Individuals with this disease are commonly managed through supplementation with synthetic thyroid hormone, with the aim of alleviating symptoms and restoring normal thyroid stimulating hormone levels. Generally this management strategy is effective and well tolerated. However, there is research to suggest that a significant proportion of hypothyroid sufferers are being inadequately managed. Furthermore, hypothyroid patients are more likely to have a decreased sense of well-being and more commonly experience constitutional and neuropsychiatric complaints, even with pharmacological intervention.

The current management of hypothyroidism follows a biomedical model. Little consideration has been given to a biopsychosocial approach to this condition. Within the chiropractic profession there is growing support for the use of a biopsychosocial-based intervention called Neuro-Emotional Technique (NET) for this population.

**Methods/Design:**

A placebo-controlled, single-blinded, randomised clinical pilot-trial has been designed to assess the influence of Neuro-Emotional Technique on a population with primary overt hypothyroidism. A sample of 102 adults (≥18 years) who meet the inclusion criteria will be randomised to either a treatment group or a placebo group. Each group will receive ten treatments (NET or placebo) over a six week period, and will be monitored for six months. The primary outcome will involve the measurement of depression using the Depression, Anxiety and Stress Scale (DASS). The secondary outcome measures to be used are; serum thyroid stimulating hormone, serum free-thyroxine, serum free-triiodothyronine, serum thyroid peroxidase auto-antibodies, serum thyroglobulin auto-antibodies as well as the measurement of functional health and well-being using the Short-Form-36 Version 2. The emotional states of anxiety and stress will be measured using the DASS. Self-measurement of basal heart rate and basal temperature will also be included among the secondary outcome measures. The primary and secondary measures will be obtained at commencement, six weeks and six months. Measures of basal heart rate and basal temperature will be obtained daily for the six month trial period, with recording to commence one week prior to the intervention.

**Discussion:**

The study will provide information on the influence of NET when added to existing management regimens in individuals with primary overt hypothyroidism.

**Trial Registration:**

ANZCTR Number: 12607000040460

## Background

Hypothyroidism is a frequently encountered clinical condition and is the most common cause of pathological hormone deficiency. Hypothyroidism is classified as either congenital or acquired, based on the time of onset. The condition is further categorised based on level of endocrine dysfunction and severity. Primary hypothyroidism follows a dysfunction of the thyroid gland itself, whereas secondary hypothyroidism results from failure in the machinery or mechanisms peripheral to the thyroid gland (eg. hypopituitarism). Overt hypothyroidism defines the clinical manifestation of the condition, and the term mild hypothyroidism is assigned to the subclinical variants of the disease [1]. The diagnosis of hypothyroidism is based on a thorough history, clinical findings and laboratory analysis. Although a deficit in thyroid hormones and their resultant action has a varying systemic influence, individuals with overt hypothyroidism frequently present with fatigue, depression, cold intolerance, weight gain, hoarseness, dry skin, bradycardia, constipation and slowed mental processing [2]. Laboratory testing in individuals with primary overt hypothyroidism typically depicts an elevated serum thyroid stimulating hormone (TSH) level, and a decrease in serum free-thyroxine (FT4) levels [3]. One of the major causes of acquired hypothyroidism is chronic autoimmune thyroiditis (Hashimoto's Disease) in which laboratory testing may reveal auto-antibodies to the thyroglobulin protein (Anti-Tg), and the thyroid peroxidase enzyme (Anti-TPO) [4,5].

The gold standard treatment for individuals with hypothyroidism is supplementation using a synthetic version (Levothyroxine Sodium) of the thyroid hormone - thyroxine [1]. Patients are typically given a supplemental dose (1.8 μg/kg in adults) of levothyroxine sodium daily for an indefinite period of time, with the aim of improving the function of the patient and restoring appropriate TSH levels [1]. Once an appropriate dose has been established, thyroid hormones levels are monitored every 6-12 months to ensure the adequacy of treatment [6]. Treatment dose may be altered based on a number of different variables; treatment efficacy; comorbid-illness; significant alterations to weight; oestrogen use; pregnancy; age [1,7].

The treatment of individuals with hypothyroidism typically follows a biomedical paradigm. There are clearly defined biochemical boundaries for what constitutes the clinical manifestation of hypothyroidism and standard algorithms to guide the commencement and progression of treatment. Several studies however have reported inadequate treatment (as determined by thyroid blood markers) in a significant proportion (20%) of individuals with 'managed' hypothyroidism [8,9]. Saravanan *et al *report that substantial numbers of hypothyroid sufferers continue to experience the plethora of hypothyroid symptoms despite demonstrating hormone levels within reference ranges [10].

Whybrow *et al *state that almost 100% of severe hypothyroid sufferers are found to have significant concurrent depression [11]. Grozinsky-Glasberg *et al *state that hypothyroid patients are more likely to have a decreased sense of well-being and more commonly experience constitutional and neuropsychiatric complaints as compared to euthyroid individuals [12]. Practitioners must take care when initiating treatment for hypothyroidism in certain populations (ischemic heart disease, adrenal cortical insufficiency) and be mindful of avoiding adverse drug interactions [1].

After an exhaustive search of the medical literature it would appear that little consideration has been given to the concept of a biopsychosocial-based management approach to this condition. Within the chiropractic profession there are anecdotal reports and case studies [13] to suggest that a biopsychosocial-based treatment approach may be applicable to hypothyroidism. This manuscript has been compiled to outline the methodology for a randomised-controlled clinical study investigating a biopsychosocial approach to primary overt hypothyroidism that adheres to the CONSORT [14] checklist in its design.

## Methods

The objectives of this research are described below:

1) To determine whether the addition of NET to concurrent management regimens can improve clinical outcomes in a sample of individuals with primary overt hypothyroidism. For Example, reducing the severity of depression scores at six weeks and six months; 2) To determine the short and medium term responses to NET via changes to the primary and secondary outcomes in this sample.

### Design

An ethics approved, registered, prospective, multivariate, placebo-controlled single-blinded, randomised, clinical pilot-trial.

### Ethics Review

The pilot-trial protocol and associated materials have been reviewed and approved by the Macquarie University Human Ethics Committee (Sydney, Australia).

### Settings

Eleven private practices located in Sydney, Australia. The private practices are situated in ten different suburbs around the Sydney region. The distribution of practice locations has been chosen in an attempt to generate a sample that will be reflective of the general population of Sydney.

### Practitioners

Twelve practitioners (five female, seven male) have agreed to donate their time to administer the treatments to the trial participants. The practitioner group consists of nine registered chiropractors, two psychologists, and one psychotherapist. Teachers of NET stipulate that all practitioners must hold a doctorate or master's level degree in the healing arts before they may learn NET. All of the care providers have a minimum of two years experience using NET, and have attained 'NET Certification' status (which denotes the highest level of proficiency recognised within the technique system). These practitioners have been chosen because of their expertise in the NET field. The chief investigator (BTB) will conduct an individual training session for each practitioner involved with the trial. In addition to the training sessions, practitioners will be given a trial reference booklet that details the specifics of the trial. Practitioners will also have phone and email access to the chief investigator at all times in the event that further questions surface following the initial briefing.

### Participants

Participants will respond via a toll-free number to advertisements placed in a variety of different media including; newspapers, magazines, websites, and flyers. An attempt will be made to disseminate hardcopy advertisements in a variety of geographical locations around Sydney. The advertisements will call for participation in a research project examining the influence of a new treatment for hypothyroidism (See Additional File 1). Respondents will be screened over the phone by the chief investigator to determine their eligibility to participate based on a set of inclusion and exclusion criteria. Eligible participants will then be sent a trial information booklet that explains the details of the trial. Participants will be given an opportunity to read through the trial information and make an informed choice as to whether or not they wish to participate in the research. One week after receiving the trial information booklet, potential participants will receive a follow-up telephone call from the chief investigator to ascertain their desire to participate, and to answer any further questions. From this point, willing participants will be sent the salient information to begin their involvement in the trial.

### Informed Consent

In addition to the trial information booklet, all participants will be issued with a copy of the informed consent form. The consent form will detail the names of the individuals involved with the study: chief investigator; supervisors/co-investigators; practitioners. Participants will also be informed that the research is being conducted by the Department of Chiropractic at Macquarie University. A brief description of the study protocols will also provided; duration of the study; outcome measures employed; adverse reactions to treatment and the associated back-up counselling services; ethics approval and contact details of the ethics officer; the right to cease participation at any time; methods for dissemination of results; complaint procedures and privacy policy. Participants will not be asked to alter or cease any existing treatment regimens. Any concurrent treatment regimens will be recorded.

### Sample

The study population will consist of at least 102 individuals with a diagnosis of overt primary hypothyroidism, who are undergoing a variety of management strategies (including no treatment), from Sydney, Australia. A justification for the sample size is provided later.

### Inclusion Criteria

For inclusion, participants will have to meet several criteria: 18 years of age or over; and previous diagnosis of primary overt hypothyroidism by a qualified medical practitioner or specialist. If respondents are being medicated for their thyroid condition, it will be required that they be on a stable dose of medication for at least six months before entering the trial. Any alteration to a participant's medication levels during the trial will be recorded. Respondents who do not meet the inclusion criteria or who meet the exclusion criteria will be excluded from the study.

### Exclusion Criteria

The exclusion criteria will comprise: less than 18 years of age; absent diagnosis of hypothyroidism; iatrogenic hypothyroidism; recent diagnosis of hypothyroidism (within the last six months); presence of co-morbid illness that can impair thyroid hormone production and metabolism; use of medications that can impair thyroid hormone production and metabolism; history of head or neck surgery/radiotherapy; history of significant head trauma; serious co-morbid illness (e.g. cancer); serious co-morbid mental illness (e.g. psychosis); pregnancy or desire to conceive within the study period; physical or mental disabilities that would prevent participants interacting physically and verbally with the care provider during the intervention.

### Withdrawals and Protocol Violations

All withdrawals and protocol violations will be recorded. As part of the trial information booklet and informed consent form, participants will be assured that they may withdraw their participation at any time without consequence or explanation. All participants who cease participation will be followed up to confirm their withdrawal. Participants will be given the option to comment on the reason for their withdrawal. If a participant does declare their reason for withdrawal, this will be recorded. Any alteration to the patient's health status or existing management regimens during the trial period will be recorded and factored into the final analysis and interpretation. This will include changes in medication/type of medication.

### Adverse Reactions

All adverse reactions will be recorded. In the published literature on NET there have been no documented adverse reactions [13,15-20]. It is not anticipated that participants will be exposed to any unnecessary risks. Due to the nature of the NET procedure/study protocol there is potential for a mild uncomfortable recall of past events in the NET treatment group. There is also potential for some mild focal soreness following the tapping procedures applied in both the NET and the placebo groups. Any participant experiencing anything more than a mild discomfort/soreness, or becoming emotionally distressed, will have their participation in the study reviewed and their potential for withdrawal assessed. In the case of any adverse reaction, the relevant help will be rendered to the participant at no charge. The primary health care provider will also be notified in the event of any significant changes/revelations in a participant's health status that are witnessed prior to commencement or during a participant's involvement in the trial.

### Randomisation and treatment allocation

Eligible participants will be randomly allocated to either the NET treatment group or the placebo (sham intervention) group. A random sequence generator will be used to produce a random sequence (1-200) with no repeats. These numbers will be recorded on individual pieces of paper and placed into envelopes which will then sealed and shuffled. After the participant has completed their initial blood test, the chief investigator will select an envelope for that participant. Participants allocated an even number will be assigned to the treatment group, and those receiving an odd number will be assigned to the placebo group.

### Blinding

The allocation of individual participants to treatment groups will be concealed to the chief investigator up until the point of a participant commencing treatment. Individual participants will not be told their group allocation until the completion of their involvement in the trial. All participants allocated to the placebo intervention group will have the opportunity to receive the NET treatment free of charge at the completion of their initial participation. No other incentives will be given for a participant's involvement or follow-up.

Any primary care providers (e.g. endocrinologist) will be blinded to the participant's group allocation. As the practitioners involved in the study will be performing both the NET treatment and the placebo intervention, it will be necessary for the group allocation to be disclosed to these practitioners. Practitioners will be instructed to perform the placebo with the same gusto as the NET treatment. The treating practitioners will have no previous role in the diagnosis or management of the participant's thyroid condition.

Group allocation will be concealed from the pathology company involved in the collection and analysis of the serum outcome measures. Statisticians involved in the analysis of the trial data will be blinded to group allocation.

### Protocol

Participants will be randomly allocated to either a treatment group (NET) or a placebo group. Both groups will receive ten interventions (either NET or placebo) during the initial 6 weeks of the trial period. Two interventions will be administered in the first four weeks followed by one intervention per week in the remaining two weeks.

### NET Protocol

Neuro-Emotional Technique was developed by Walker [21] in 1985, and can be summarised as a 15-step, multi-modal intervention that incorporates principles from several health disciplines, including; chiropractic, biology, cognitive behavioural psychology, and Traditional Chinese Medicine (TCM)[22]. A brief description of the NET procedure is described below. It is important to note that the procedure is based on many different theories. Some of these theories have been well researched, while others have not undergone the same scientific scrutiny.

The NET procedure is specifically designed to detect and remove a specific type pathophysiological/syndromal pattern called a Neuro Emotional Complex (NEC). Walker postulates that the presence of NECs has a negative influence on an individual's physiology, and that by removing NECs using NET, physiological homeostasis may be promoted [21].

Walker defines the NEC as a subjective maladaption syndrome adopted by the organism in response to a real or perceived threat to any aspect of its survival [21]. Walker states that the NEC/syndromal pattern will contain the aspects listed below:

1)A facilitated or inhibited muscle; 2) A specific active Meridian Access Point (MAP); 3)An imbalance in a meridian, and an active pulse point or MAP point; 4) A specific emotion; 5) A cathected and often recallable memory picture (Snap Shot) of a past significant emotional event; 6) A conditioned response with a resistance to extinction, and a predisposition for stimulus generalization; 7) A vulnerability to suppression, repetition-compulsion and reaggravation/restimulation causing a cyclical reinforcement; and 8) A specific subluxation or sequence of spinal subluxations.

The protocol uses manual muscle testing (MMT) to aid the practitioner in finding and removing NECs. A muscle test in which the individual is unable to withstand a standardised test pressure is termed an inhibited/weak (w) test. A muscle test in which an individual is capable of withstanding a standardised test pressure from the practitioner is termed a facilitated/strong (s) test [21]. Strong and weak test outcomes are used to provide feedback for the practitioner throughout the protocol. The specific steps of the protocol and the muscle testing outcomes at the various steps are outlined in the appendix (See Additional File 2) [21].

There are two methods used in NET to detect the presence of an NEC. The two types are defined by Walker as a Mind-Entry (ME) and a Body-Entry (BE). The ME is used to detect the presence of an NEC by having the patient either think of a situation/memory/feeling or by verbalising specific statements [21,23]. The BE method of detecting an NEC involves having the patient or practitioner touch specific cutaneous areas on the body recognised in TCM to be related to the presenting condition. NET is based on the notion that there will be demonstrable changes in a patient's physiology when a salient ME or BE is employed. Manual muscle testing (MMT) is used to demonstrate the physiological change associated with the NEC [21]. For convenience, the anterior deltoid muscle test is most commonly used during the NET procedure [21].

Walker states that NET engages the energetic system of the body as described by TCM. In TCM, it is thought that there are 12 main channels throughout the body that carry vital energy - termed *chi*. These channels are called meridians. Disruption to the flow of chi along a meridian channels is believed to result in disease and ill health [24]. It is postulated that elements of the NEC are stored within this energetic network. Walker opines that an NEC stored within the meridian system can be accessed via cutaneous points associated with the meridians (See Additional File 3 &4) [21] and MMT [21].

According to the Five Element Theory of TCM, each of the meridians is associated with specific emotions. For example, the liver meridian is said to be associated with the emotion of Anger [25]. An exaggerated experience of emotion (either too much, too little, or prolonged experience of a specific emotion) can cause disruption to the free flow of chi through the meridians. The normal experience of emotion is not pathological, but extremes of emotion are thought to cause disharmony within the meridian system [24,25]. Walker and others have expanded upon this concept and created tables to which NET practitioners can refer to during the NET protocol (See Additional File 5) [21]. It is thought that NECs are created during times (past or present) in which a person is experiencing physiological or meridian deficit [21].

The NET procedure is therefore designed to find NECs that are purported to be having a negative influence on an individual's physiological and energetic system. The energetic imbalance accompanying the NEC will have a related emotion and a recallable memory picture/snap shot that is associated with that emotion. Using the NET protocol in combination with MMT, the specific emotion and its associated snap shot are detected.

It is thought that the NEC may fail to resolve under normal circumstances and can become a conditioned response that is subject to restimulation/reaggravation in reaction to a variety of different stimuli. For this reason, NET is thought to aid in the extinction of classically conditioned responses to traumatic emotional stimuli [18].

Walker states that there will be specific spinal regions that will require treatment for each type of meridian imbalance (See Additional File 5).

Once an appropriate BE or ME has been found, a specific meridian imbalance, a specific emotion, and a recallable snap shot have been displayed, a practitioner can proceed with the corrective phase of the technique. The corrective procedure involves having the individual think of the original snap shot and the associated emotion, while at the same time holding the corresponding MAP point (See Additional File 4)[21]. The practitioner then applies a light, bilateral tapping to specific spinal regions on breathing [21].

The original snap shot and the original BE or ME are then tested to assess the adequacy of the treatment.

During the intervention phase, the trial practitioners will seek to find and remove any NECs that may be present in the trial participants using NET.

It is important to note that the version of NET to be used in this study (12-step protocol) does not represent the NET system in its entirety. The 12 Step protocol utilises Steps 1-11, and Step 15. The originators of NET champion the combined use of manual therapy, nutritional supplementation and homeopathic treatment in addition to addressing the psychosocial aspects of a patient's presentation [21]. For research purposes, the initial three aspects mentioned above (manual therapy, nutrition, and homeopathy) and steps 12-14 of the NET protocol will be omitted to limit the number of confounding variables, and ensure a more accurate interpretation of the results. The protocol to be used in this research will be similar to that employed in previous research papers describing NET as a treatment modality [16,26-31]. Karpouzis et al published a similar protocol for the use of NET for Attention Deficit Hyperactivity Disorder in an open access journal in 2009 [16].

### Placebo Protocol

The placebo protocol will align clearly with the NET protocol however the purported therapeutic components of the NET procedure will be removed and replaced with innocuous steps (See Additional File 6.)

### Measures

The standard guidelines for the assessment of individuals with thyroid dysfunction will be used by the primary care providers to establish the diagnosis of hypothyroidism [1]. The primary and secondary outcome measures (DASS, SF-36v2 and Serum Measures) will be applied pre-intervention to obtain baseline data, then reapplied at six weeks (post-intervention) and six months (study conclusion). Measurement of basal heart rate and basal temperature will be applied for each day of the six month study period, and will commence one week prior to the research intervention.

### Primary Outcome Measures

Significant proportions of individuals with 'managed' hypothyroidism continue to experience many of the symptoms associated with the overt, untreated version of the condition [1]. Depression is a common symptom associated with primary overt hypothyroidism [1]. Hypothyroid patients are more likely to experience constitutional and neuropsychiatric complaints as compared to euthyroid individuals [12]. For this reason, the primary outcome will involve the measurement of Depression using of the Depression Anxiety Stress Scale (DASS). The DASS is an instrument for measuring the negative states of depression, anxiety and stress. It is constructed of three self-report scales that enable researchers and clinicians to more concisely define, understand and measure these three clinical states. Each of the three scales contains 14 items that assess different aspects of depression, anxiety and stress [32].

The questionnaire is used to determine negative emotional states versus negative emotional traits. The DASS is based on a dimensional concept of psychological disorder. For this reason, the DASS is not used to allocate patients to specific diagnostic categories. This concept is different to other classificatory systems such as the Diagnostic and Statistical Manual of Mental Disorders (DSM) and International Classification of Diseases (ICD).

All items are rated on a four-point Likert scale from 0 - 3. Scores are determined according to how often particular symptoms were experienced in the past week, with high scores indicating a higher frequency of symptoms. Depression represents a sum of scores from 14 items within the DASS pertaining to depression [32]. States of dysphoria, hopelessness, devaluation of life, self-deprecation, anhedonia, inertia and a lack of interest and involvement are assessed by the 'depression' specific questions within the DASS. Normative data for the Depression scale of the DASS describes a mean of 6.34 with a standard deviation of 6.97. Clinically, depression scores between 0-9 are considered normal. Scores of 10-13 and 14-20 indicate mild and moderate states of depression respectively. Severe and extreme states of depression are defined by scores between 21-27 and 28+ respectively [32].

The DASS questionnaire will be issued to each participant at the specified times; study commencement, six-weeks and six-months. The results will be collected and forwarded to the chief investigator for compilation and scoring using relevant scoring algorithms and software [32].

Improvements in the negative emotional state of depression will be represented by decreased scores by respondents at 6 weeks and at 6 months. The associated anxiety and stress scores from the DASS will be included in the secondary outcome measures.

### Secondary Outcome Measures

The secondary outcome measures (from serum analysis) are listed in Table 1. All blood collection and analysis will be performed by the commercial pathology laboratory DHM. The pathology company DHM provides pathology services to general practitioners, medical specialists, nursing homes, private hospitals and researchers [33].

**Table 1:** Serum Secondary Outcome Measures

Test Name	Abbreviation	Reference Range
Thyroid Stimulating Hormone	TSH	0.40 - 4.00 mU/L
Free Thyroxine	FT4	9.0 - 19 pmol/L
Free Triiodothyronine	FT3	2.6 -6.0 pmol/L
Thyroglobulin auto-antibodies	Anti-Tg	0 - 80 IU/ml
Thyroid Peroxidase auto-antibodies	Anti-TPO	0 - 120 IU/L

Participants will be issued with a blood sample request form that can be used at any DHM collection centre. Blood samples will be obtained at the beginning of the trial, then again at the six week and six month points. Participants will be instructed to provide a blood sample that will be used to measure a variety of thyroid related indicators (See Table 1). There will be no specification regarding timing or fasting for the sample collection. The initial blood sample will completed one week prior to the first treatment in both groups. Blood samples will be spun in gel tubes at the collection centre before being couriered in cold storage to the central DHM laboratory for analysis. At the central laboratory, the ARCHITECT^® ^assay kits (Chemiluminescent Microparticle Immunoassay) will be used to measure the TSH, FT3, FT4 in the serum samples (Abbott Laboratories, Abbott Park, IL, USA). Immulite^® ^2500 will be used to quantify levels of anti-thyroid peroxidase antibodies and auto-antibodies to thyroglobulin in serum. The Immulite^® ^2500 method involves a solid-phase, enzyme labelled, chemiluminescent sequential immunometric assay (Siemens, Los Angeles, CA, USA). All results will be forwarded to the chief investigator for monitoring, compilation and analysis.

It is anticipated that the majority of participants will be receiving treatment in the form of L-Thyroxine supplementation. For this reason, serum measures of TSH, serum FT4, serum FT3 should fall within normal reference ranges (See Table 1) provided there are no major alterations to this existing treatment strategy. In adequately treated participants, the addition of the experimental intervention should not influence serum thyroid hormone levels. However, in individuals who are not being medicated, or in individuals who are being under-treated upon entry into the trial, a decrease in TSH measurements and an increase in FT4 and FT3 measurements may be expected during the course of the trial.

In individuals who are being over-treated, elevated FT4 and FT3 levels and decreased TSH measurements will be demonstrated at baseline. It is expected that there will be no change in the serum measures in this subgroup.

It is unclear how the autoimmune markers will respond to the addition of NET to any existing treatment regimens.

In addition to the serum measures, participants will fill out the SF-36v2 and the additional items (anxiety and stress) of the DASS.

The SF-36v2 health survey is a questionnaire used to assess outcomes in eight different domains: role limitations because of physical or emotional problems, physical and social functioning, bodily pain, perception of mental and general health, and vitality [34].

Improvements in functional health and well-being as measured by the SF-36v2 will be depicted by increased scores by the respondents at six weeks and at six months. Deterioration in functional health and well-being will be revealed by a decrease in SF-36v2 scores.

Two additional secondary outcome measures are to be completed and recorded by participants at home for each day of the six month trial period are listed below:

Basal Heart Rate (BHR)

Basal Temperature (BT)

After signing up to take part in the trial, participants will be given a home diary, digital thermometer and instructions on how to record their BHR and BT. Participants will be instructed to make an effort to perform these measurements at the same time each day, and ideally before getting out of bed in the morning. For BHR measurements, participants will be instructed to find their radial pulse and then record the number of beats that occur in one minute and record this figure in the diary provided.

Due to the chronotropic effect of the thyroid hormones on heart rate, untreated or undertreated individuals will demonstrate lower heart rates at baseline. It is anticipated that if NET therapy has a positive influence, there may be a trend over time toward slight increases in heart rate.

Basal temperature recording will be performed using a digital thermometer placed under the axilla. The thermometer will be placed under the axilla for approximately two minutes and the resultant temperature reading recorded.

Due to the thermogenic action of thyroid hormones, it is hypothesised that unmedicated or under-treated participants will experience an increase over time in basal temperature that may approach normal ranges of basal temperature (35.5 - 37.0°C) with the addition of NET treatment [35]. Adequately treated individuals should display normal basal temperatures at baseline that remain unperturbed by any additional treatment.

The secondary measures of basal heart rate and temperature measurement to be employed in this research study are commonly used by thyroid specialists as a crude measure of thyroid performance that is adjunctive to the hormone assays [36-38].

The recording of BHR and BT will begin one week prior to the initial intervention and continue for each day of the clinical trial.

### Power analysis and sample size justification

A (one-sided) power analysis indicated 51 subjects per group will yield an 80% chance of detecting a decrease in depression scores of at least four points in treatment compared to control using a standard deviation of eight based on simulation. Clinically, a four point reduction in depression scores, as defined by the DASS, would represent a meaningful reduction in depressive states. A four point decrease may indicate that an individual has gone from expressing moderate to mild states of depression or mild to normal expression of depressive states [32].

### Statistical Analysis

Two-sample t-tests will be used to compare change from baseline for NET treatment versus control at each of six weeks and six months for the primary outcome measure. Linear mixed-effects models will be used examine change over time in the treatment and placebo groups for the basal temperature and basal heart rate groups as well as the other secondary outcome measures. A significance level of 5% will be used throughout the analysis. Because the analyses will be seen as hypothesis generating, no adjustment for multiple testing will be used. The R software program [39] will be used to conduct the analyses. Complete case analysis will be employed for participants who strictly followed the trial protocol. Sensitivity analysis will be used for withdrawals, missing data and participants who violate the study protocol eg. changes in medication during the study period. This will be done using imputation techniques including last value carried forward, and multiple imputation.

## Discussion

We have presented the design and protocol for a placebo-controlled, single-blinded RCT that will investigate the influence of a biopsychosocial-based treatment approach for primary overt hypothyroidism. The participants of this study will have NET treatment added to any existing management regimens. Anecdotal evidence from within the chiropractic profession suggests that NET treatment may be of benefit for individuals with hypothyroidism. Completion of this trial will help to describe the influence of NET treatment when combined with any existing management strategies for individuals with primary overt hypothyroidism.

### Status of the trial and expected achievements

Start date: September 2006; Finish date of recruitment: March 2010; Finish date of follow-up: September 2010; Dissemination of results: Jan 2011.

## Abbreviations

ANTI-TG: thyroglobulin auto-antibodies; ANTI-TPO: thyroid peroxidase auto-antibodies; BE: Body Entry; BHR: Basal Heart Rate; BT: Basal Temperature; CONSORT: Consolidated Standard of Reporting Trials; DASS: Depression Anxiety Stress Scale; FT3: free triiodothyronine; FT4: free thyroxine; ME: Mind entry; MMT: Manual Muscle Testing; NEC: Neuro Emotional Complex; NET: Neuro-Emotional Technique; S: strong; SF-36 V2: Short Form-36 Version Two; TCM: Traditional Chinese Medicine; TSH: thyroid stimulating hormone; W: weak.

## Competing interests

The chief investigator BTB, RB, and PG declare that they have no competing interests. HP is a part-time employee of the ONE Foundation, which is a not-for-profit organization which aims to support research into NET.

## Authors' contributions

BTB, RB, and HP conceived the study, participated in its design and drafted the manuscript. PG advised on the statistical analysis and also drafted the manuscript. All authors read and approved the final manuscript.

## Supplementary Material

Additional file 1 **Sample of an advertisement used to recruit participants for the clinical pilot-trial**. Click here for file

Additional file 2 **15 steps of the NET protocol**. Click here for file

Additional file 3 **Pulse points used in the NET protocol**. Click here for file

Additional file 4 **Meridian Access Points used in the NET protocol**. Click here for file

Additional file 5 **Simplified NET master chart used in the NET protocol**. Click here for file

Additional file 6 **Placebo protocol used in the pilot trial**. Click here for file
