# Supplementary material for: The influence of a biopsychosocial-based treatment approach to primary overt hypothyroidism: a protocol for a pilot study
Source: Trials. 2010 Nov 15;11:106. doi: 10.1186/1745-6215-11-106 (PMC2992059; doi:10.1186/1745-6215-11-106)
Supplement: Additional file 1 — Sample of an advertisement used to recruit participants for the clinical pilot-trial. [file 1745-6215-11-106-S1.PDF]

Do You Have Hypothyroidism?

Are you interested in trying a
NEW treatment?

You are invited to participate in a research study investigating a drug-free treatment for hypothyroidism. The study will be conducted by the Department of Health and Chiropractic at Macquarie University.

The study will examine the influence of a NEW chiropractic technique on individuals with hypothyroidism.

Participants will be offered a FREE course of chiropractic treatment at one of the various research clinics around Sydney.

For more information contact Benjamin T. Brown on

Ph: 1800 800 490 any time from Monday to Sunday or
Email: benjamin.brown@students.mq.edu.au

ABN 90 952 801 237 | CRICOS Provider No 00002J
