# Supplementary material for: The influence of a biopsychosocial-based treatment approach to primary overt hypothyroidism: a protocol for a pilot study
Source: Trials. 2010 Nov 15;11:106. doi: 10.1186/1745-6215-11-106 (PMC2992059; doi:10.1186/1745-6215-11-106)
Supplement: Additional file 2 — 15 steps of the NET protocol. [file 1745-6215-11-106-S2.DOC]

**Additional File 2:** NET Protocol

**For NET Mind Entry begin at Step 1**

**For NET Body Entry**

(A) Find body entry – **(w)**

(B) Counter with Emotional points – **(s)**

(C) Disconnect and begin Step 1

1. Step 1 – Find “issue” - **(w)**
2. Step 2 – Counter “issue” to a meridian access point (MAP) or pulse point - **(s)**

**(s)** Step 3 – Validate the emotion - **(w)**

**(s)** Step 4 – Find whose emotion – for someone else’s emotion use the word “sensitive to” - **(w)**

**(s)** Step 5 – Create a concept using the “issue” and the emotion – then add a “because or why” - **(w)**

**(s)** Step 6 – “Lets check the concept of the original event where there was (insert emotion from Step 3) because (insert simplified ‘because’ from step 5)” - **(w)**

**(s)** Step 7- Index for time - **(w)**

**(s)** Step 8 – Patient finds original Snap Shot – “Where were you and what were you doing?” - **(w)**

**(w)** Step 9 – Counter Snap Shot to MAP found in step 2 - **(s)**

Step 10 – Have the patient hold the emotional points, the MAP or pulse point from step 2, and the original snap shot from Step 8. Stimulate the specific vertebral levels for the salient MAP with three phases of respiration or alternatively hold the emotional points and the salient pulse point and breathe in and out for a few minutes.

**(s)** Step 11 – Retest the original Snap Shot - **(s)**

**(s)** Step 12 – (*Optional*) “Test any other emotions”

**(s)** Step 13 – Test homeopathic BMI with corrected MAP/pulse point

NB – If Steps 12 or 13 are strong go to Step 15

**(w)** Step 14 – Add the candidate remedy under patient’s tongue and retest BMI with MAP/pulse point - **(s)**

**(s)** Step 15 – Retest starting entry (Step A for body entry or Step 1 for mind entry) - **(s)**
