# Supplementary figures and images for: The influence of a biopsychosocial-based treatment approach to primary overt hypothyroidism: a protocol for a pilot study
Source: Trials. 2010 Nov 15;11:106. doi: 10.1186/1745-6215-11-106 (PMC2992059; doi:10.1186/1745-6215-11-106)

**Additional File 3:** Pulse Points


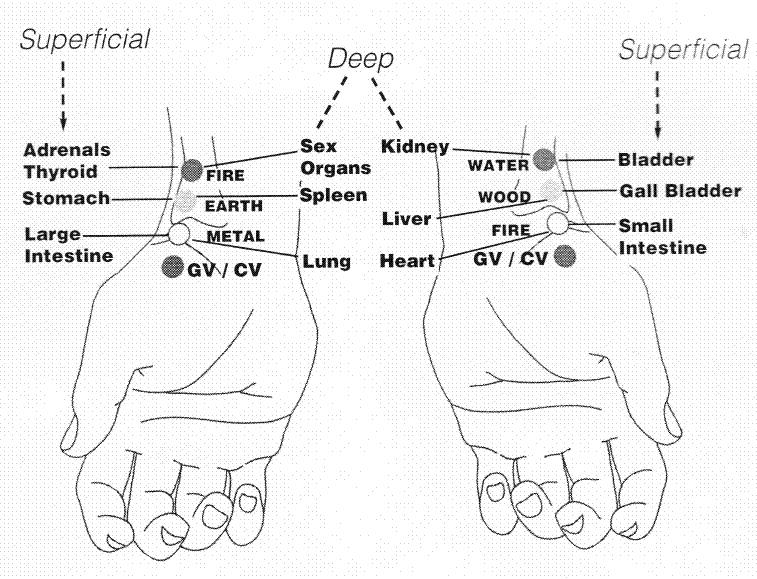

Supplement: Additional file 3 — Pulse points used in the NET protocol. [file 1745-6215-11-106-S3.DOC]

**Additional File 4:** Meridian Access Points (MAPs)


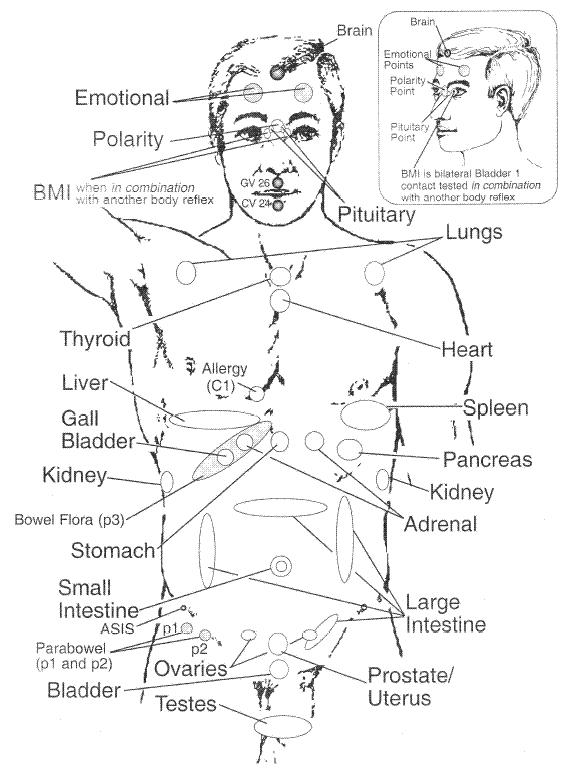

Supplement: Additional file 4 — Meridian Access Points used in the NET protocol. [file 1745-6215-11-106-S4.DOC]

**Additional File 5:** Simplified NET Master Chart


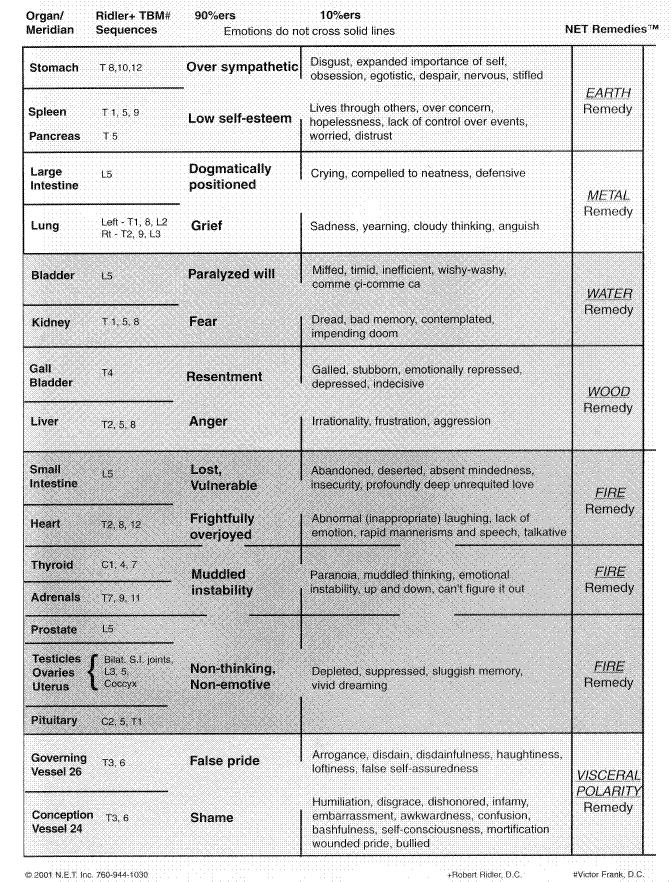

Supplement: Additional file 5 — Simplified NET master chart used in the NET protocol. [file 1745-6215-11-106-S5.DOC]
