# Supplementary material for: The influence of a biopsychosocial-based treatment approach to primary overt hypothyroidism: a protocol for a pilot study
Source: Trials. 2010 Nov 15;11:106. doi: 10.1186/1745-6215-11-106 (PMC2992059; doi:10.1186/1745-6215-11-106)
Supplement: Additional file 6 — Placebo protocol used in the pilot trial. [file 1745-6215-11-106-S6.DOC]

**Additional File 5:** Placebo Protocol Flow Chart

**Placebo Diagnostic Phase**

**For NET Body Entry** - (A) Find body entry (B) Counter with Emotional points (C) Disconnect

(s) 1 – Find “issue” - (w)

1. 2 – Counter “issue” to a meridian access point (MAP) or pulse point - (s)

**Placebo Corrective Phase**

3 – Have the patient sit with the arms comfortably by their side. Stimulate the right inferior border of the scapula five times while the patient breathes normally.
